# I like it when my partner holds my hand: development of the Responses and Attitudes to Support during Pain questionnaire (RASP)

**DOI:** 10.3389/fpsyg.2014.01027

**Published:** 2014-09-19

**Authors:** Charlotte Krahé, Yannis Paloyelis, Chiara F. Sambo, Aikaterini Fotopoulou

**Affiliations:** ^1^Department of Psychology, Institute of Psychiatry, King's College LondonLondon, UK; ^2^Department of Neuroimaging, Institute of Psychiatry, King's College LondonLondon, UK; ^3^Department of Neuroscience, Physiology and Pharmacology, University College LondonLondon, UK; ^4^Research Department of Clinical, Educational and Health Psychology, University College LondonLondon, UK

**Keywords:** social support, acute pain, questionnaire, attachment style, pain behavior, healthcare professionals

## Abstract

Social support can have beneficial effects on psychological and physiological well-being. During acute bodily pain, however, the effects of social support on pain are mixed. This variability may be due to the multifaceted nature of both pain and social support, as well as individual differences. In this paper, we present the development, psychometric assessment, and initial validation of the first self-report measure designed to address this variability in the general population; the Responses and Attitudes to Support during Pain questionnaire (RASP). The RASP includes questions on social support from the romantic partner as well as healthcare professionals (HCPs) and addresses different types of social support and pain responses. The development and validation of the RASP comprised four studies. In Study 1, a preliminary RASP version was completed by 179 healthy individuals regarding any type of acute pain. In Study 2, the reduced RASP was completed by 256 women with experiences of menstrual pain. Principal component analysis indicated a 22-item solution with five underlying dimensions reflecting *General Partner Support, Emotional Support from HCPs, Anxiety in the Context of HCPs, Pain Behaviors during Partner Support*, and *Distraction by the Partner*. Construct validity was assessed using a measure of adult attachment style. The RASP showed good validity and test-retest reliability. In Study 3, the 5-factor model received initial support through confirmatory factor analysis in a new sample of 120 individuals with recent musculoskeletal pain. Study 4 provided additional validation of the RASP in a sample of 180 individuals responding in reference to acute back pain. Overall, the RASP is a valid and reliable measure for assessing individual differences in attitudes and responses to social support in relation to acute pain.

## Introduction

Pain is a multifaceted psychological state, arising in response to actual or potential tissue damage (International Association for the Study of Pain, 1994) and is frequently experienced within a social context. However, while the cognitive and affective modulation of pain have received much attention (e.g., Villemure and Bushnell, 2002; Salomons et al., 2004; Lumley et al., 2011), social contextual factors, such as social support, have been studied chiefly in chronic pain samples (Newton-John, 2002; Leonard et al., 2006) and far less in acute pain samples. Social support is also a multidimensional construct (Barrera, 1986), commonly divided into emotional support (e.g., reassurance), instrumental support (e.g., tangible help) and informational support (e.g., advice; Schaefer et al., 1981). The type of social support provided has been shown to differentially affect pain (e.g., Chambers et al., 2002; Brown et al., 2003; Jackson et al., 2005), and its effects interact with the source of social support (Dakof and Taylor, 1990; Masters et al., 2007). In one study, back pain patients preferred emotional and instrumental support over informational support when a friend provided support, while they rated instrumental support as most helpful from doctors (Masters et al., 2007). Emotional support was most unhelpful from spouses (Masters et al., 2007). In contrast, another study found that cancer patients perceived emotional support as more helpful than unhelpful from spouses, informational support as helpful from other cancer patients, physicians and nurses, and instrumental support as most helpful from nurses (Dakof and Taylor, 1990). While both studies highlight that the effects of social support type depend on the source of support, they (a) used chronic or clinical samples and (b) found differing results regarding emotional support from spouses, highlighting the need for further research.

In the last decade, experimental studies have investigated the causal mechanisms underlying the social modulation of pain by using experimentally-induced pain (reviewed in Krahé et al., 2013). These studies have manipulated different types of support, ranging from simply priming socially supportive themes (Younger et al., 2010; Eisenberger et al., 2011) to manipulating the behavior of present support providers (e.g., Brown et al., 2003; Jackson et al., 2005). They have also found interactions between type and source of support; for example, holding another person's hand (vs. an object) reduced pain only when it was a partner's and not a stranger's hand (Master et al., 2009).

In addition, studies have noted the critical role of individual differences in predicting how social support affects individuals' experiences of pain. In particular, adult attachment style, a personality construct relating to both the perception of social interactions and pain, moderated the effects of social support on pain in experimental settings (Sambo et al., 2010; Wilson and Ruben, 2011; Hurter et al., 2014). However, given the relatively small samples and the kind of social variables that can be studied under experimental control, the results of these studies are difficult to generalize and transfer to different acute pain conditions. Instead, suitable theory-based and ecologically-valid measures of the impact of social support are needed that take into account the multiple dimensions of both pain and the social context in which it occurs. The present research developed and tested a measure of individual differences in attitudes and responses to a range of sources and types of social support in the context of acute pain, which may ultimately be useful for individually tailoring the provision of social support in acute pain settings.

There is currently no comprehensive theoretical framework that can accommodate the multifaceted and interactive nature of source and type of social support and their effects on acute pain. Indeed, most theories describing the association between social support and pain stem from chronic pain research (e.g., operant conditioning, Fordyce, 1976; the communal coping model of pain catastrophising, Sullivan et al., 2000; intimacy models, Cano and Williams, 2010) and address certain facets of pain, notably communicative pain behaviors. Our measure combined insights from these different theories to develop an instrument that reflects the multiple underlying dimensions of the social modulation of acute pain.

While the complex relations between social support and chronic pain have been studied using suitable questionnaires (e.g., Kerns et al., 1985; McWilliams et al., 2009), there is a lack of relevant measures and studies focusing on acute pain in the general population, in which the romantic partner and healthcare professionals are frequent sources of support. Hence, we developed (Study 1) and provided the psychometric evaluation (Studies 2–4) of a self-report questionnaire assessing attitudes and responses to social support from the partner and health care professionals in relation to the experience of acute bodily pain; the Responses and Attitudes to Support during Pain questionnaire (RASP).

## Study 1: development of the RASP in the general population

### Aims

In Study 1, we aimed to (a) construct items for the preliminary RASP, and (b) use principal component analysis to test the initial component structure of the RASP and reduce the number of items.

### Measures

#### Construction of RASP items and the preliminary RASP

The RASP was designed to assess attitudes and responses to social support in relation to the experience of acute bodily pain. To this end, we conducted a thorough review of the clinical and experimental social modulation of pain literature and consulted extant reviews (e.g., Payne and Norfleet, 1986; Leonard et al., 2006). This revealed the diversity in theoretical and methodological approaches inherent in the literature and mentioned in the introduction (see also Krahé et al., 2013). Importantly, none of the existing theoretical frameworks could explain this diversity, particularly as most theoretical frameworks stem from research on chronic and not acute pain. We thus opted for using a combination of theoretical perspectives to inform the construction of the questionnaire which could, when adopted in future studies, enable novel theoretical insights into the social modulation of acute pain. Although, of course, it was not possible to construct a measure that included all possible facets of the social modulation of acute pain, we endeavored to include a set of sources and types of social support and a range of pain-related responses most frequently encountered in the literature and associated with stronger effects (Krahé et al., 2013). The links to their respective theoretical (mostly chronic pain) models and empirical findings are indicated below.

We chose “partner,” “friend,” and “healthcare professionals” (henceforth HCPs) as sources of social support. While such relationship and healthcare contexts may not apply to all members of the population or instances of acute pain, our selection was made due to the well-known frequency and relevance of contact with these sources during pain as well as a host of research into social support from these three sources (Dakof and Taylor, 1990; Masters et al., 2007). Regarding HCPs, doctors, dentists and nurses were selected specifically as they are part of primary care services and therefore should be familiar points of contact to most individuals.

Furthermore, six types of social support were included to cover a range of support behaviors. The types of support chosen were those most regularly covered in conceptualizations of social support or assessed within the social context of pain. First, “social presence” (also termed passive support; Brown et al., 2003) was included, as it has been investigated in clinical studies on chronic pain (e.g., Flor et al., 1995) and in experimental studies on acute pain (Brown et al., 2003; Montoya et al., 2004), including in the context of the communal coping model of pain catastrophizing (e.g., Sullivan et al., 2004; Vervoort et al., 2008, 2011). Related to more active and emotional support, “social reassurance” and “empathy” (relevant to operant conditioning and intimacy models; Fordyce, 1976; Cano and Williams, 2010) were incorporated as relevant supportive behaviors by others (Krahé et al., 2013). “Touch” was also added given its recognized role as an indicator of an affiliative and supportive attitude that is embodied rather than verbal and can have analgesic effects (Master et al., 2009). “Offering help” was included as a variable of instrumental support (see Schaefer et al., 1981). Lastly, social distraction was added because of the recognized influence of distraction on pain in social as well as non-social settings (e.g., Villemure and Bushnell, 2002; Jackson et al., 2005; Jackson, 2007). Informational support was not incorporated as we felt this variable may be subject to differences in knowledge and information availability in different social groups that we would not be able to measure.

Finally, four different pain-related responses were included. “Pain intensity” and “pain distress and anxiety” were chosen to reflect sensory and affective-motivational dimensions of pain, respectively (as in Melzack, 1987). Further, “worry of pain consequences” and “pain behaviors” were incorporated to address theoretical perspectives such as the communal coping model of pain catastrophizing (Sullivan et al., 2000) which place emphasis on these facets. Although some pain facets are moderately correlated (Labus et al., 2003), they can also be dissociated (Rainville et al., 1999), and thus these four dimensions were included separately. In addition, desirability of support was added to assess whether there were individual differences in attitudes regarding certain types of support from different sources over and above the potential effects of such types and sources of support on the other four pain-related responses (akin to Dakof and Taylor, 1990; Masters et al., 2007). Thus, the RASP was constructed such that it could be related to a range of pain experiences. Study 1 did not restrict the type of pain experience. In Studies 2 and 3, samples were selected to maximize the availability of experiences of certain types of acute pain. In Study 4, participants were not selected on the basis of specific pain experiences but were asked to think of a certain type of acute pain.

Each RASP item included one source of support, one type of support, and one facet of pain. After creating the 90 items (3 sources of support × 6 types of support × 5 pain-related facets), it became apparent that not only was the length of the questionnaire unfeasible but several combinations of dimensions were not relevant. For example, “touch” (e.g., hand-holding, hugging) did not seem to apply to interactions with HCPs. Thus, HCP touch items for all pain-related facets and similar inapplicable combinations were omitted, resulting in 57 items which were presented to participants. Twenty-nine items included the partner as support provider, 10 pertained to a friend, and 18 related to HCPs. Items were phrased to include both positive (37 items) and negative (20 items) responses, i.e., referring to decreases and increases in pain, respectively, to avoid biasing participants. Example items were, “*I like it when my partner holds my hand when I am in pain*” (Partner, Touch, Desirability), “*When a doctor/dentist has understanding for my pain, it seems to make the pain less*” (Doctor/Dentist, Empathy, Pain Intensity), and “*When I am in pain, I prefer that my partner does not ask me what he or she can do to help*” (Partner, Offering Help, Desirability). Items were constructed using different but semantically similar words to denote the pain-related responses (e.g., discomfort for anxiety), and Cronbach's alpha was used to ensure that the items indeed captured the same underlying constructs (see Principal component analysis results). All items were presented with a five-point rating scale, with 1 labeled “*never true,”* 2 “*true some of the time*,” 3 “*true half of the time*,” 4 “*true most of the time*,” and 5 “*true all the time*.”

#### Items assessing the relationship with social support providers

As relationship quality has been shown to influence the effects of social support on pain and threat (Coan et al., 2006), the RASP was presented with several items assessing the relationship with the different sources of social support. Participants were asked to indicate whether they were thinking of a current partner, previous partner or close family member (if they had never been in a relationship) when responding to the “partner” items. Furthermore, participants were asked to rate their “partner's” and “friend's” level of habitual empathy on a scale from 1 (*not empathic at all*) to 5 (*extremely empathic*), the level of closeness with their “partner” and “friend” on a scale from 1 (*not close at all*) to 5 (*extremely close*) and the degree of happiness in their relationship with their “partner” and “friend” on a scale from 1 (*not at all happy*) to 5 (*extremely happy*). In addition, they indicated the length of friendship for the close friend they were thinking of while completing the “friend” items.

#### Demographic information

Participants were asked to state their gender, age, nationality, country of residence, and whether or not they had a history of chronic pain.

### Participants and procedure

One hundred and ninety participants from the general population completed the study. Participants had to be fluent in English to take part; no other inclusion or exclusion criteria were applied. Although items pertained to the “partner,” participants with and without a current partner were recruited for this initial development phase (as in McWilliams et al., 2009). Eleven individuals were classed as outliers on several of the RASP variables (see Principal component analysis) and were excluded from analyses. Thus, the final sample consisted of *N* = 179 individuals from the general population. Full demographic details are presented in Table 1.

**Table 1:** **Sample characteristics for Studies 1–4**.

	**Study 1 (*N* = 179^a^, 34 items)**		**Study 2 (*N* = 256, 22 items)**	**Study 3 (*N* = 120, 22 items)**	**Study 4 (*N* = 180, 22 items)**
Gender	47 (26.40%) men; 131 (73.60%) women		All female	38 (31.67%) male; 82 (68.33%) female	93 (51.7%) male; 87 (48.3%) female
Age	*M* = 23.93 years (*SD* = 8.95)		*M* = 24.22 years (*SD* = 5.72)	*M* = 28.85 years (*SD* = 11.11)	*M* = 33.75 years (*SD* = 10.13)
Nationality	81.9% British, 14.1% other European, 4% rest of the world		64.1% British, 17.6% other European, 9.0% Asian, 9.3% rest of the world	78.3% British, 11.7% other European, 10% rest of the world	97.6% USA, 2.4% rest of the world
Country of residence	93.8% UK, 4.5% other European country, 1.7% rest of the world		92.2% UK or other English-speaking country, 7.8% rest of the world	93% UK, 6.7% rest of the world	100% USA
Person thought of when answering partner questions	48% current partner; 23.5% past partner; 28.5% family member		–	–	–
Length of friendship	*M* = 8.41 years (*SD* = 6.72)		–	–	–
Length of romantic relationship	–		*M* = 37.29 months (*SD* = 42.73)	*M* = 64.10 months (*SD* = 93.78)	*M* = 90.57 months (*SD* = 103.32)
HCP visit in relation to pain			32% visited a HCP	71.7% visited a HCP	70.6% visited a HCP in the last year, of which 38.58% indicated that primary reason for visit was pain
Time since injury	–		–	69.2% less than 1 year; 12.5% 1–2 years; 18.3% 2 years +	–
Chronic pain (lasting longer than 3 months) and mood disorder	5.6% chronic pain (no question on mood disorder)		15.2% chronic pain; 12.9% mood disorder; of these, 3.9% both chronic pain and mood disorder	18.3% chronic pain; 5.8% mood disorder; one participant (0.83%) both chronic pain and mood disorder	31.7% chronic pain; 11.7% mood disorder
Frequency of pain; 1 (*never*) to 5 (*all of the time*)	–		*M* = 3.76 (*SD* = 0.81)	–	–
Pain intensity; 0 (*no pain*) to 10 (*pain as intense as you can imagine*)	–		*M* = 6.11 (*SD* = 1.92)	*M* = 5.59 (*SD* = 2.07)	23.3% were in pain when completing questionnaire; *M* = 4.00 (*SD* = 1.77)
Perceived empathy; 1 (*not empathic at all*) to 5 (*extremely empathic*)	Partner	*M* = 3.37 (*SD* = 0.94)	*M* = 3.54 (*SD* = 1.04)	*M* = 3.30 (*SD* = 1.14)	*M* = 3.44 (*SD* = 0.98)
	Friend	*M* = 3.44 (*SD* = 1.06)	–	–	–
Closeness; 1 (*not close at all*) to 5 (*extremely close*)	Partner	*M* = 3.95 (*SD* = 0.99)	*M* = 4.35 (*SD* = 0.75)	*M* = 3.99 (*SD* = 0.92)	*M* = 4.10 (*SD* = 0.92)
	Friend	*M* = 3.87 (*SD* = 0.85)	–	–	–
Relationship happiness; 1 (*not at all happy)* to 5 (*extremely happy)*	Partner	*M* = 3.85 (*SD* = 0.99)	*M* = 4.14 (*SD* = 0.85)	*M* = 3.98 (*SD* = 0.91)	*M* = 3.91 (*SD* = 1.00)
	Friend	*M* = 4.00 (*SD* = 0.72)	–	–	–

a *One person did not provide demographic information but did complete the RASP and is therefore included in the main analyses*.

Several aspects of the procedure were identical for all studies reported in this paper and are thus summarized here. In all studies, the RASP was presented online. In Studies 1–3, participants were contacted by university circular e-mails and online advertisements that contained the link to the questionnaire. In Study 4, participants were recruited via the online platform Amazon Mechanical Turk. Online questionnaires were designed using www.selectsurvey.net software and all questions were forced-entry items. Participants were first required to tick an informed consent box to proceed to the main questionnaire. To ensure anonymity, assess test-retest reliability (Study 2), and determine whether any participants had completed the questionnaire more than once, participants entered a self-generated ID. In the present study, contrary to Studies 2–4, questions about demographic details were presented at the end of the questionnaire. The overall completion time was approximately 15–20 minutes. Ethical approval for this research was obtained from King's College London Psychiatry, Nursing and Midwifery Research Ethics Subcommittee.

### Plan of statistical analyses

#### Principal component analysis

The data was first examined for multivariate outliers on the RASP variables by calculating Mahalanobis distances (evaluated against chi square statistics at 57 degrees of freedom and *p* < 0.001); outliers were excluded from analyses.

Principal component analysis (PCA) was employed to test the initial component structure of the RASP and exclude weak items. We selected PCA because the primary goal was to reduce the number of variables (Floyd and Widaman, 1995; Tabachnick and Fidell, 2007). As these were initial analyses, we did not calculate or interpret scale scores. The following decisions presented here also applied to Study 2.

The data's suitability for PCA was assessed using the Kaiser-Meyer-Olkin measure, the anti-image correlation matrix, and Bartlett's test of sphericity (see Field, 2009). We decided how many components to retain by considering three criteria: (1) eigenvalues > 1 (Kaiser's criterion; Kaiser, 1960), (2) examining the scree plot, and (3) conducting a parallel analysis (Monte Carlo PCA for parallel analysis; Watkins, 2000).

As components were theoretically expected to be connected, we used oblique rotation (direct oblimin) to help with their interpretation (Tabachnick and Fidell, 2007; Field, 2009). The pattern matrix containing the component loadings was interpreted, and item loadings below 0.40 were considered poor and were suppressed (Tabachnick and Fidell, 2007).

### Results

#### Principal component analysis results

The data were found to be suitable for PCA. With all 57 items, the Kaiser-Meyer-Olkin measure of sampling adequacy was KMO = 0.846, Bartlett's test was significant at *p* < 0.001, and examination of the anti-image correlation matrix revealed that all KMO values for individual variables were ≥0.567 (see Field, 2009, for guidelines regarding acceptable values).

Examination of eigenvalues, scree plot, and the results of the parallel analysis for the initial PCA indicated that six components should be retained. A sequence of PCA was conducted, successively eliminating poor items (those loading on more than one component or no component, with low loadings, i.e., falling below <0.55 (Comrey and Lee, 1992), or whose deletion improved the Cronbach's alpha of the corresponding component), and testing the component structure with each reduced item set. The final analysis yielded a six-component solution, accounting for 64.06% of the variance, on the basis of 32 items relating to the partner and health care professionals (items pertaining to friends were not retained in the final solution). Items with component loadings and corrected item-total correlations are presented in Table 2 and correlations among the components are displayed in Table 3.

**Table 2:** **Items with component loadings and corrected item-total correlations for Studies 1 and 2**.

		**Study 1 (*N* = 179, 34 items)**		**Study 2 (*N* = 256, 22 items)**	
		**Component loading**	**Corrected item-total correlation**	**Component loading**	**Corrected item-total correlation**
**COMPONENT 1: *GENERAL PARTNER SUPPORT***					
Having my partner there when I am in pain, makes me feel the pain less.	Partner, Social Presence, Intensity (P_SP_I)	0.866	0.770	0.822	0.725
If my partner holds my hand when I am in pain, I experience the pain as less intense.	Partner, Touch, Intensity (P_T_I)	0.860	0.785	0.726	0.701
If my partner offers to help me when I am in pain, I experience the pain as somewhat less intense.	Partner, Offering Help, Intensity (P_OH_I)	0.829	0.805	0.829	0.834
If my partner offers me some reassurance, it decreases my pain somewhat.	Partner, Social Reassurance, Intensity (P_SR_I)	0.819	0.718	0.654	0.737
If my partner offers to help me when I am in pain, I find the pain less unpleasant.	Partner, Offering Help, Anxiety (P_OH_A)	0.736	0.708	0.779	0.654
I am less worried about the potential consequences of my pain if my partner hugs or cuddles me when I am in pain.	Partner, Touch, Pain Consequences (P_T_C)	0.721	0.747	0.680	0.647
I am less worried about the consequences of my pain if my partner offers to help me when I am in pain.	Partner, Offering Help, Pain Consequences (P_OH_C)	0.717	0.719	0.703	0.660
When I am in pain I feel less discomfort if my partner is with me.	Partner, Social Presence, Anxiety (P_SP_A)	0.685	0.626	0.737	0.609
**COMPONENT 2: *ANXIETY IN CONTEXT OF HCPs***					
I feel more anxious if a nurse tries to reassure me about my pain.^*^	Nurse, Social Reassurance, Anxiety (N_SR_A)	0.801	0.656	0.669	0.466
If a doctor/dentist or nurse offers practical help when I am in pain, it tends to make me more anxious about my pain.^*^	Doctor/Dentist/Nurse, Offering Help, Anxiety (DDN_OH_A)	0.738	0.647	0.834	0.606
I do not like it if a doctor/dentist or nurse tries to help me to stand or reach for my things when I am in pain.^*^	Doctor/Dentist/Nurse, Offering Help, Desirability (DDN_OH_DES)	0.672	0.416	–	–
I do not feel comfortable when a doctor/dentist shows empathy for my pain.^*^	Doctor/Dentist, Empathy, Desirability (DD_E_DES)	0.663	0.516	–	–
It makes me feel more apprehensive about my pain if a doctor/dentist or a nurse encourages me to talk about something else to distract me.^*^	Doctor/Dentist/Nurse, Distraction, Anxiety (DDN_D_A)	0.657	0.534	0.734	0.541
If a doctor/dentist tries to reassure me when I am in pain it makes me think there is something to worry about.^*^	Doctor/Dentist, Social Reassurance, Anxiety (DD_SR_A)	0.612	0.551	0.805	0.631
**COMPONENT 3: *DISTRACTION (STUDY 1)/DISTRACTION BY PARTNER* (STUDIES 2–4)**					
It helps relieve anxiety for my pain if my partner makes me think about other things.	Partner, Distraction, Anxiety (P_D_A)	0.832	0.794	0.763	0.780
It gives me some relief from pain if my partner talks to me about other things in order to distract me.	Partner, Distraction, Intensity (P_D_I)	0.806	0.689	0.755	0.751
I find it nice when my partner tries to distract me from my pain by engaging me in other activities or topics of conversation.	Partner, Distraction, Desirability (P_D_DES)	0.800	0.714	0.902	0.816
It eases my pain if a doctor/dentist of a nurse talks to me to take my mind off the pain.	Doctor/Dentist/Nurse, Distraction, Intensity (DDN_D_I)	0.687	0.704	–	–
I appreciate it if a doctor/dentist or nurse talks to me about something pleasant to distract me from my pain.	Doctor/Dentist/Nurse, Distraction, Desirability (DDN_D_DES)	0.551	0.599	–	–
**COMPONENT 4: *EMOTIONAL SUPPORT FROM HCPs***					
I find it nice if a nurse is understanding and caring when I am in pain.	Nurse, Empathy, Desirability (N_E_DES)	0.844	0.699	−0.847	0.715
I like it when a doctor/dentist reassures me about the pain I go through.	Doctor/Dentist, Social Reassurance, Desirability (DD_SR_DES)	0.824	0.712	−0.883	0.774
I prefer it if a nurse is reassuring when I am in pain.	Nurse, Social Reassurance, Desirability (N_SR_DES)	0.794	0.757	−0.902	0.789
It soothes me when a nurse shows me empathy when I am in pain.	Nurse, Empathy, Anxiety (N_E_A)	0.740	0.705	−0.737	0.647
If a nurse does not pay much attention to my pain, I feel as if the pain gets worse.	Nurse, Empathy, Intensity (N_E_I)	0.499	0.434	–	–
**COMPONENT 5: *PAIN BEHAVIORS IN CONTEXT OF PARTNER***					
If my partner does not care about my pain, I tend to exaggerate my pain.^*^	Partner, Empathy, Pain Behavior (P_E_PB)	0.773	0.613	–	–
If my partner tries to talk to me about other things in order to distract me from my pain, I tend to exaggerate my pain (Study 1)/express my pain more (Studies 2–4).^*^	Partner, Distraction, Pain Behavior (P_D_PB)	0.768	0.653	−0.736	0.514
If my partner tries to reassure me about my pain, I tend to exaggerate my pain in talking about it (Study 1)/express my pain by talking about it more (Studies 2–4).^*^	Partner, Social Reassurance, Pain Behavior (P_SR_PB)	0.755	0.639	−0.792	0.513
If my partner gets me to rest and helps me with my jobs when I am in pain, I tend to exaggerate the pain (Study 1)/express the pain more (Studies 2–4).^*^	Partner, Offering Help, Pain Behavior (P_OH_PB)	0.749	0.477	−0.817	0.585
**COMPONENT 6: *DESIRABILITY OF PARTNER SUPPORT***					
When I am in pain, I prefer that my partner does not ask me what he or she can do to help.^*^	Partner, Offering Help, Desirability (P_OH_DES)	0.803	0.427	–	–
When I am in pain, I prefer my partner not to be with me.^*^	Partner, Social Presence, Desirability (P_SP_DES)	0.545	0.411	–	–
I want my partner to reassure me when I am in pain.	Partner, Social Reassurance, Desirability (P_SR_DES)	−0.535	0.570	–	–
I like it if my partner shows empathy when I am in pain.	Partner, Empathy, Desirability (P_E_DES)	−0.492	0.502	–	–

Items with

* *are negatively phrased*.

**Table 3:** **Component correlation matrix for the final component solution derived from PCA in *N* = 179 individuals with non-specific acute pain (Study 1)**.

***RASP component***	***General Partner Support***	***Emotional Support from HCPs***	***Anxiety in Context of HCPs***	***Pain Behaviors in Context of Partner***	***Distraction by Partner***	***Desirability of Partner Support***
*General Partner Support*	1	0.390	−0.005	0.205	0.407	−0.238
*Emotional Support from HCPs*		1	−0.030	0.195	0.274	−0.151
*Anxiety in Context of HCPs*			1	0.168	−0.049	0.164
*Pain Behaviors in Context of Partner*				1	−0.009	−0.031
*Distraction by Partner*					1	−0.070
*Desirability of Partner Support*						1

Component 1 accounted for 28.05% of the variance and included eight items, all pertaining to support from the partner and including a variety of support behaviors. Therefore, this component was labeled *General Partner Support*. Cronbach's alpha for this component was α = 0.923.

Component 2 accounted for 12.07% of the variance and comprised six items. All items featured HCPs as the support providers, and the majority included anxiety as the facet of pain. All items on this component were negatively phrased, so that a higher score on each item indicated e.g., an increase in anxiety. Given the emphasis on anxiety, this component was named *Anxiety in Context of HCPs*. Cronbach's alpha for this scale was α = 0.791.

Component 3 explained 7.84% of the variance and consisted of five items. This was the only component to contain items on social support from both the partner and HCPs. All items included distraction as the type of social support. Therefore, the component was labeled *Distraction*. Cronbach's alpha was α = 0.872.

Component 4 described 6.85% of the variance and consisted of five items, all pertaining to support from HCPs, predominantly nurses. The types of social support were empathy and social reassurance; thus, this component was named *Emotional Support from HCPs*. Cronbach's alpha was α = 0.846.

Component 5 accounted for 4.94% of the variance and included four items, all concerning the partner as the source of social support and pain behaviors as the pain response. As with the *Anxiety in Context of HCPs* scale, items on this component addressed negative pain responses, i.e., a higher score denoted more pain behaviors. This component was named *Pain Behaviors in Context of Partner*. Cronbach's alpha was α = 0.774.

Lastly, component 6 accounted for 4.30% of the variance and consisted of four items. All items referred to the partner and desirability of support. This was the only component to include positively and negatively phrased items. The component was named *Desirability of Partner Support*. Internal consistency was α = 0.691.

In sum, Study 1 reported the initial development of the RASP in a sample from the general population. The internal consistency of the six components was good to excellent, denoting that the items, despite their variability in wording (see Construction of RASP items and preliminary RASP), seemed to describe the same underlying dimension.

## Study 2: psychometric evaluation of the RASP in women with menstrual pain

### Aims

The aims of this study were four-fold: first, to further develop and psychometrically evaluate the RASP in a group with recent concrete pain experiences, we selected a more homogeneous sample of individuals with experiences of a common and specific type of pain, namely menstrual pain, who also reported being in a romantic relationship (to enhance the applicability of the partner items). The second aim was to reduce the length of the RASP further to avoid repetition and also to add clarifications to individual items to increase the RASP's utility. Third, to assess the psychometric properties of the RASP, two aspects of reliability and validity were evaluated. Test-retest reliability was examined in a subsample of participants. Menstrual pain is well-suited to investigate reliability across time as there is a natural retest point at the next menses. Further, construct validity was explored by studying associations between the RASP and a measure of adult attachment style. The fourth aim was to explore relationships between demographic variables and RASP subscales.

### Measures

#### Revised version of the responses and attitudes to support during pain questionnaire (RASP)

Two steps were undertaken to further improve the RASP prior to its administration to participants in this study. In the first step, six individuals completed the 32-item RASP derived in Study 1 and subsequently reported any issues they had encountered in responding to items. Two changes were made on the basis of this feedback: (1) four items containing the word “exaggerated” were changed to make them sound more neutral; for example, the item “*If my partner does not care about my pain, I tend to exaggerate my pain*” was changed to “*If my partner does not care about my pain, I tend to express my pain more*”, and (2) a definition of empathy was included, in which empathy was defined “as the sense of knowing or understanding the experience and feelings of another individual” (adapted from Goubert et al., 2005). In the second step, to address the lack of empathy and pain intensity combination for doctors/dentists in the initial RASP, we added the item, “*I feel less pain if the doctor, dentist or nurse explains what is causing the pain in an empathic way*” (Doctor/Dentist/Nurse, Empathy, Pain Intensity). In order to include one item on informational support from HCPs, we also added the item, “*I feel less pain if the doctor, dentist or nurse tells me that pain is expected*” (Doctor/Dentist/Nurse, Information, Pain Intensity). Thus, the revised RASP consisted of 34 items; 32 items from the preliminary 57-item RASP plus the two new items. The source of support was either the partner (19 items) or HCPs (15 items), and 12 items were phrased to have negative pain-related responses.

#### Items assessing the relationship with social support providers

As in Study 1, participants provided information on the quality of the relationship with their partner (see Items assessing the relationship with social support providers in Study 1 for details).

#### Items assessing participants' pain experience per se

As participants in this study had specific acute pain experiences, they were asked to rate how much pain they were currently experiencing/had most recently experienced in reference to their menstrual pain on a scale from 0 (*no pain*) to 10 (*pain as intense as you can imagine*), and how often they experienced menstrual pain during their menses. Participants were also asked whether they had visited a HCP for their menstrual pain.

#### Experiences in close relationships revised questionnaire (ECR-R; Fraley et al., 2000)

The ECR-R is a 36-item self-report questionnaire assessing adult attachment style. Participants in Study 3 also completed this measure but details are presented here. Half the ECR-R items pertain to attachment anxiety, e.g., “*I'm afraid that I will lose my partner's love*.” The other items pertain to attachment avoidance, e.g., “*I prefer not to be too close to romantic partners*.” Items are presented with a scale from 1 (*strongly disagree*) to 7 (*strongly agree*). The ECR-R yields scores on both attachment anxiety and attachment avoidance dimensions, with lower scores denoting greater attachment security and higher scores denoting greater attachment insecurity (14 items are reverse-scored). Both ECR-R scales have been found to be moderately positively correlated (e.g., Sibley et al., 2005). The ECR-R has been extensively used to measure adult attachment style (see Ravitz et al., 2010) and is well-validated (Sibley et al., 2005).

We used the ECR-R to assess construct validity in this study (validation using further measures was undertaken in Study 4; see below). We chose adult attachment style because we sought to validate the RASP with a construct strongly related to both the perception of social support (see, e.g., Collins and Feeney, 2004, for links between adult attachment style and social support) *and* pain (see Meredith, 2013, for a review of the literature demonstrating the association between adult attachment style and pain). In addition, adult attachment style is an *inter*personal construct, specifically capturing attitudes and responses regarding the perceived availability and responsiveness of others in times of threat, such as pain. In particular, individuals high in attachment anxiety exhibit strong dependency and reaching out to others during threat (Bartholomew and Horowitz, 1991). In relation to the RASP, a positive correlation was thus expected between attachment anxiety and positively phrased RASP scales, i.e., that higher attachment anxiety would be associated with more positive attitudes and responses to social support. In contrast, individuals high in attachment avoidance strive to maintain independence and are characterized by their mistrust of others (Hazan and Shaver, 1987). Low trust in others to supply care has been linked to a greater intention to delay seeking care in patients with possible acute coronary syndromes (Sullivan et al., 2009). In addition, avoidant individuals are less likely to turn to their support network for help or advice than secure or anxious individuals (Wallace and Vaux, 1993). We therefore expected a negative correlation between attachment avoidance and positively phrased RASP scales, i.e., that higher avoidance scores would be linked to negative attitudes and responses to social support in relation to pain.

#### Demographic information

The same questions as in Study 1 were asked (see Study 1). Participants were also asked whether or not they had a history of depression.

### Participants and procedure

Two additional inclusion criteria were specified for this sample (see Study 1 for overall recruitment procedures). Participants were included only if they were women who (a) experienced moderate-severe menstrual pain, and (b) were in a romantic relationship at the time of taking part. The online questionnaire was completed by *N* = 256 women. Of these, 23 women filled in the questionnaire a second time approximately four weeks after their first participation (i.e., the length of an average menstrual cycle). Their first visit data was included in the main analysis, while their second visit data was used only to assess test-retest reliability. Full demographic details for the *N* = 256 women used in the main analysis are displayed in Table 1. The mean age of the *n* = 23 subsample was *M* = 23.26 years (*SD* = 4.69) at Time 1, and their mean relationship length was *M* = 25.61 months (*SD* = 18.00) at Time 1 and *M* = 26.78 months (*SD* = 18.07) at Time 2. The procedure was very similar to that outlined in Study 1 apart from a slight change in order and the addition of the ECR-R. Participants first provided demographic details and then completed the RASP and ECR-R in a fixed order. Within the ECR-R, items were presented in a randomized fashion (as suggested by http://internal.psychology.illinois.edu/~rcfraley/measures/ecrritems.htm).

### Plan of statistical analyses

#### Principal component analysis

As in Study 1, the data was subjected to principal component analysis (PCA) to further reduce the length of the RASP, explore the RASP component structure in a specific acute pain sample, and examine similarities between the component structures in Studies 1 and 2.

#### Scale reliability

RASP scale scores were computed by taking the mean of the items loading on each component (DiStefano et al., 2009). Test-retest reliability was evaluated in a subsample of participants by computing mean intraclass correlation coefficients (ICCs) in a two-way random-effects model (where “time” and “participant” were the factors). Values below 0.40 were considered poor, values between 0.40 and 0.59 as fair, 0.60–0.74 as good, and values above 0.75 as excellent (Fleis et al., 2003).

#### Construct validity

Construct validity was explored by examining correlations between the RASP scales and a measure of adult attachment style, the ECR-R [see Experiences in close relationships revised questionnaire (ECR-R; Fraley et al., 2000)]. As our hypotheses pertained to each ECR-R scale separately and the ECR-R scales are generally moderately positively correlated (Sibley et al., 2005; *r* = 0.387, *p* < 0.001 in the present sample), partial correlations were calculated to assess the association of each scale individually with the RASP (akin to methods used previously; Fraley et al., 2011).

#### The relationship between sample characteristics and RASP scales

Demographic variables and items pertaining to the relationship with social support providers were correlated with RASP scale scores to evaluate the role of demographic factors and relationship quality in responses and attitudes to social support during pain. As the RASP scales derived in this sample were exploratory, bivariate correlations of the RASP scales with the continuous variables (age, length of relationship, partner empathy, partner closeness, and relationship happiness), and one-way analyses of variance (ANOVAs) were carried out separately for each RASP scale to assess differences on the categorical variables (history of chronic pain, history of depression).

### Results

#### Principal component analysis

With all 34 variables, i.e., the 32 items retained in the final solution in Study 1 plus the two new variables [see Revised version of the responses and attitudes to support during pain questionnaire (RASP)], the data showed very good sampling adequacy (Kaiser-Meyer-Olkin measure = 0.862; values ≥ 0.697 in the anti-image correlation matrix) and correlations between variables existed in the data (Bartlett's test was significant at *p* < 0.001), demonstrating that the data was suitable for PCA. As components were correlated in Study 1, oblique rotations were applied.

As in Study 1, a series of PCA was conducted, eliminating poor items and examining the component structure at each step. The final 22-item five-component solution accounted for 66.75% of the variance. Items with component loadings and corrected item-total correlations are presented in Table 2.

Component 1 accounted for 30.20% of the variance and included eight items, all pertaining to support from the partner. As items on this component were identical to the *General Partner Support* component in Study 1, this component was also labeled *General Partner Support*. Cronbach's alpha was α = 0.904.

Component 2 accounted for 13.38% of the variance and included four items, all pertaining to support from HCPs. As the items loading on this component were all contained in the *Emotional Support from HCPs* component in Study 1, this component was again named *Emotional Support from HCPs*. Cronbach's alpha was α = 0.872.

Component 3 accounted for 9.56% of the variance and contained four items, again referring to HCPs. As the pain facet in all four items was anxiety, and all items were also contained in the *Anxiety in Context of HCPs* components in Study 1, the component was again called *Anxiety in Context of HCPs*. Higher scores on this component denoted more anxiety. Cronbach's alpha was α = 0.758.

Component 4 accounted for 7.22% of the variance and consisted of three items, all referring to the partner as source of support. In addition, all items included pain behaviors as the pain facet and were contained in the component *Pain Behaviors in Context of Partner* in Study 1. Therefore, this component was also named *Pain Behaviors in Context of Partner*. As with Component 3, higher scores on this scale referred to increased pain behaviors. Cronbach's alpha was α = 0.711.

Lastly, Component 5 accounted for 6.40% of the variance and included three items in which the type of social support was distraction and which featured in the component *Distraction* in sample 1. However, contrary to sample 1, the items included only the partner as source of support and thus the component was named *Distraction by Partner*. Cronbach's alpha was α = 0.844.

#### Scale reliability

Mean RASP scale scores and correlations among scales are presented in Table 4. Higher scores on *Anxiety in Context of HCPs* and *Pain Behaviors in Context of Partner* scales denoted increased anxiety and pain behaviors, while higher scores on the *General Partner Support, Emotional Support from HCPs* and *Distraction by Partner* scales signified decreases in pain facets.

**Table 4:** **Mean scale scores for the RASP (standard deviation (SD) represents ± 1 SD from the mean) and Pearson correlations among RASP scales for Studies 2, 3, and 4**.

**RASP scale**	**Mean (SD)**			***Emotional Support from HCPs***			***Anxiety in Context of HCPs***			***Pain Behaviors in Context of Partner***			***Distraction by Partner***		
**Study**	**2**	**3**	**4**	**2**	**3**	**4**	**2**	**3**	**4**	**2**	**3**	**4**	**2**	**3**	**4**
*General Partner Support*	2.96 (0.97)	2.95 (0.97)	3.07 (1.03)	0.325^**^	0.523^**^	0.237^**^	0.251^**^	0.325^**^	0.197^**^	0.288^**^	0.374^**^	0.425^**^	0.537^**^	0.588^**^	0.720^**^
*Emotional Support from HCPs*	3.64 (0.99)	3.41 (1.03)	3.47 (1.16)	1	1	1	−0.051	0.152	−0.036	0.112	0.173	0.192^**^	0.134^*^	0.432^**^	0.193^**^
*Anxiety in Context of HCPs*	1.74 (0.76)	1.81 (0.81)	1.78 (0.97)				1	1	1	0.242^**^	0.379^**^	0.436^**^	0.129^*^	0.224^*^	0.063
*Pain Behaviors in Context of Partner*	1.80 (0.80)	1.86 (0.82)	2.16 (0.97)							1	1	1	0.163^**^	0.195^*^	0.297^**^
*Distraction by Partner*	3.48 (1.01)	3.30 (1.11)	3.50 (0.98)										1	1	1

** *Correlation is significant at the 0.01 level (2-tailed)*.

* *Correlation is significant at the 0.05 level (2-tailed)*.

*For the Anxiety in Context of HCPs and Pain Behaviors in Context of Partner scales, high scores denote more anxiety and more pain behaviors, respectively; for the remaining scales high scores denote less anxiety, less pain intensity etc*.

For the test-retest sample, ratings of partner's level of empathy, closeness with partner and relationship happiness were significantly correlated at time points 1 and 2, *r* = 0.840 (*p* < 0.001), *r* = 0.541 (*p* = 0.008), and *r* = 0.649 (*p* = 0.001), respectively, indicating that relationship quality remained stable over time. Mean intraclass correlation coefficients (ICCs) were ICC = 0.764 for *General Partner Support*, ICC = 0.602 for *Emotional Support from HCPs*, ICC = 0.676 for *Anxiety in Context of HCPs*, ICC = 0.520 for *Pain Behaviors in Context of Partner* and ICC = 0.524 for *Distraction by Partner* scales. Therefore, test-retest reliability was fair to excellent for the five RASP scales (Fleis et al., 2003).

#### Construct validity

Twenty-six participants were missing 1–3 ECR-R items due to a problem with the online questionnaire. For these participants, items were imputed with the mean of the remaining items on the appropriate ECR-R scale. Mean ECR-R scores were *M* = 2.65 (*SD* = 1.13) for attachment anxiety (20–30th percentile, Fraley, R.C., personal communication, 2011) and *M* = 2.39 (*SD* = 1.02) for attachment avoidance (30–40th percentile). Cronbach's alphas were α = 0.915 for attachment anxiety and α = 0.927 for attachment avoidance, indicating excellent internal consistencies.

Partial correlations are presented in Table 5. *General Partner Support*, *Emotional Support from HCPs*, and *Distraction by Partner* scales, capturing positive attitudes and responses to social support in relation to pain, were positively correlated with attachment anxiety and negatively related to attachment avoidance, as predicted given the need for closeness and reassurance in the former and the self-reliance and discomfort with closeness characterizing the latter (Hazan and Shaver, 1987). In addition, the *Anxiety in Context of HCPs* scale was positively associated with attachment anxiety, meaning that higher attachment anxiety related to more *Anxiety in Context of HCPs*, potentially indicating an overlap between attachment anxiety and general social anxiety (Cassidy et al., 2009). Attachment avoidance was not correlated with *Anxiety in Context of HCPs*, which could be due to the employment of deactivation strategies in avoidant individuals, including the inhibition of threat- or anxiety-related thoughts in the context of pain (Wilson and Ruben, 2011). No significant correlations were found between the *Pain Behaviors in Context of Partner* scale and either attachment style dimension.

**Table 5:** **Partial correlations between attachment anxiety, attachment avoidance and the five RASP scales in Studies 2, 3, and 4**.

**RASP Scale**	**Study 2 (*n* = 256 with menstrual pain)**		**Study 3 (*n* = 116 with musculoskeletal pain)**		**Study 4 (*n* = 177 thinking of acute back pain)**	
	**ECR-R anxiety**	**ECR-R avoidance**	**ECR-R anxiety**	**ECR-R avoidance**	**ECR-R anxiety**	**ECR-R avoidance**
*General Partner Support*	0.269^**^	−0.254^**^	0.271^**^	−0.393^**^	0.221^**^	−0.437^**^
*Emotional Support from HCPs*	0.228^**^	−0.239^**^	0.299^**^	−0.314^**^	0.018	−0.205^*^
*Anxiety in Context of HCPs*	0.165^**^	−0.029	0.134	0.020	0.196^*^	0.071
*Pain Behaviors in Context of Partner*	0.091	0.054	0.128	0.035	0.271^**^	−0.107
*Distraction by Partner*	0.149^*^	−0.162^**^	0.087	−0.117	0.184^*^	−0.525^**^

** *Correlation is significant at the 0.01 level (2-tailed)*.

* *Correlation is significant at the 0.05 level (2-tailed)*.

*Correlations between ECR-R Anxiety and RASP scales control for ECR-R Avoidance while correlations between ECR-R Avoidance and RASP scales control for ECR-R Anxiety. For Anxiety in Context of HCPs and Pain Behaviors in Context of Partner scales, higher scores denote an increase in anxiety and pain behaviors*.

#### The relationship between sample characteristics and RASP scales

One-way analyses of variance (ANOVAs) revealed no differences between individuals with and without chronic pain [*F*_(1, 254)_ = 0.592, *p* = 0.442 for *General Partner Support*; *F*_(1, 254)_ = 2.047, *p* = 0.154 for *Emotional Support from HCPs*; *F*_(1,254)_ = 0.390, *p* = 0.533 for *Anxiety in Context of HCPs*; *F*_(1, 254)_ = 0.283, *p* = 0.595 for *Pain Behaviors in Context of Partner*; *F*_(1, 254)_ = 0.419, *p* = 0.518 for *Distraction by Partner*], or with and without depression [*F*_(1, 254)_ = 0.774, *p* = 0.380 for *General Partner Support*; *F*_(1, 254)_ = 2.734, *p* = 0.099 for *Emotional Support from HCPs*; *F*_(1, 254)_ = 0.227, *p* = 0.634 for *Anxiety in Context of HCPs*; *F*_(1, 254)_ = 0.838, *p* = 0.361 for *Pain Behaviors in Context of Partner*; *F*_(1, 254)_ = 2.248, *p* = 0.135 for *Distraction by Partner*] on any of the RASP scale scores, justifying their joint consideration in the sample.

Examining the relationship between demographic variables and RASP scales, there were significant negative correlations of age with *General Partner Support* (*r* = −0.277, *p* < 0.001), *Anxiety in Context of HCPs* (*r* = −0.184, *p* = 0.003), and *Distraction by Partner* scales (*r* = −0.278, *p* < 0.001), suggesting that attitudes and responses to partner support and anxiety around HCPs became less pronounced with age. Length of relationship was significantly albeit weakly related only to the *Distraction by Partner* scale (*r* = −0.176, *p* = 0.005), indicating that distraction was viewed as less beneficial the longer the duration of participants' relationship had been. Lastly, when RASP scale scores were correlated with the relationship quality items, only the association between relationship happiness and *Pain Behaviors in Context of Partner* was significant (*r* = −0.174, *p* = 0.005), indicating that greater relationship happiness was linked to fewer pain behaviors.

Supporting the choice of menstrual pain as a relevant type of acute pain, participants rated the intensity of their pain as moderate and indicated that they experienced pain moderately frequently during their menses. Choosing a sample of women with experiences of menstrual pain thus maximized the availability of a recent, concrete, and identifiable pain experience to be thought of when completing the RASP. Thirty-two percent of participants had visited a HCP for their menstrual pain. As this meant that the majority of participants had not had contact with HCPs relating to their menstrual pain, an aim for Study 3 was to select a sample with more exposure to HCPs to increase the pertinence of the HCP items.

In brief, this study yielded a 22-item version of the RASP representing five underlying dimensions: (1) *General Partner Support*, (2) *Emotional Support from HCPs*, (3) *Anxiety in Context of HCPs*, (4) *Pain Behaviors in Context of Partner*, and (5) *Distraction by Partner*. This 5-component model was established in women with experience of menstrual pain who reported being in a relationship. The RASP showed good to excellent internal consistency and good construct validity and test-retest reliability. Given these results, Study 3 was designed to provide initial confirmation of this model in a new sample of individuals in romantic relationships with recent experiences of a different type of acute pain.

## Study 3: initial confirmation of the component structure of the final version of the RASP in individuals with musculoskeletal pain

### Aims

Study 3 aimed to examine the fit of the five-component RASP model identified in Study 2 in a novel dataset using confirmatory factor analysis (CFA). Due to low rates of HCP contact in the menstrual pain sample, musculoskeletal pain was selected as the type of acute pain because it is likely to require contact with HCPs. Given the different type of pain in this study, CFA was used as an initial check that the model derived from Study 2 was acceptable. In addition, construct validity was examined by again correlating RASP scales with the two adult attachment style dimensions.

### Measures

#### Final version of the responses and attitudes to support during pain questionnaire (RASP)

The 22-item RASP derived from Study 2 was administered to participants. Fourteen items pertained to partner support and eight pertained to support from HCPs; seven items were negatively phrased.

#### Items assessing the relationship with social support provider

As in Studies 1 and 2, participants rated the quality of their relationship with their current partner (see Study 1 for details).

#### Items assessing participants' pain experience per se

Akin to Study 2, participants in Study 3 were asked to rate how much pain they were currently experiencing/had most recently experienced in reference to their musculoskeletal pain (see Study 2 for details). Furthermore, participants indicated whether they had visited a HCP for their musculoskeletal pain and how long ago their injury had occurred.

#### Experiences in close relationships revised (ECR-R; Fraley et al., 2000)

See Experiences in close relationships revised questionnaire (ECR-R; Fraley et al., 2000) in Study 2 for details.

#### Demographic information

The same questions as in Study 2 were asked.

### Participants and procedure

Participants were included, irrespective of gender, if they had recently suffered from broken/fractured bones, muscle sprain/strains/tears, tendonitis or torn ligaments/tendons, and were in a romantic relationship at the time of taking part (see Study 1 for overall recruitment procedures). The sample consisted of *N* = 120 individuals. Comprehensive sample characteristics are displayed in Table 1.

### Planned statistical analyses

#### Confirmatory factor analysis and construct validity

The data was first examined for univariate and multivariate outliers by examining leverage indices. Outliers were defined as being five times greater than the sample average leverage value (Brown, 2006).

The raw data was subjected to confirmatory factor analysis (CFA) to assess model fit. Following the results in Study 2, we specified a model with five factors, namely *General Partner Support, Emotional Support from HCPs, Anxiety in Context of HCPs, Pain Behaviors in Context of Partner* and *Distraction by Partner*, with the items comprising each scale in Study 2 being assigned to the corresponding factor in the CFA. CFA was used to examine whether the derived model showed an acceptable fit to the new data, rather than to alter the existing model (i.e., a purely confirmatory rather than exploratory aim), and therefore no *post-hoc* corrections to the model were applied. As components were correlated in Studies 1 and 2, latent variables were allowed to be correlated in the model. Model fit was evaluated using the chi-square test of model fit, root mean square error of approximation (RMSEA), comparative fit index (CFI), Tucker-Lewis index (TLI), standardized root mean square residual (SRMR), and by assessing the magnitude of correlation between indicators and their latent variables. To determine whether the 5-factor model provided a substantially better fit to the data than a more parsimonious model, we also ran a model specifying only one factor (with all indicators being assigned to this factor) and compared this “baseline” model fit with our 5-factor model. Construct validity was assessed using the same measure of attachment style as in Study 2.

#### The relationship between sample characteristics and RASP scales

As in Study 2, demographic and relationship quality variables were correlated with RASP scale scores. In addition, one-way ANOVAs were carried out to assess whether there were any differences on the categorical variables (history of chronic pain, history of depression, gender). These analyses were followed up by a series of multiple linear regression analyses for each RASP scale separately, entering sex (dummy coded) and age in step 1, and length of relationship, partner empathy, partner closeness, and relationship happiness in step 2.

### Results

#### Confirmatory factor analysis

No univariate or multivariate outliers were identified and thus all cases were included in the CFA. Five latent variables (*General Partner Support, Emotional Support from HCPs, Anxiety in Context of HCPs, Pain Behaviors in Context of Partner*, and *Distraction by Partner*) were specified to relate to the 22 RASP indicators (items) derived from Study 2. “P_OH_I” (see column with abbreviations in Table 2) was used as a marker indicator for the *General Partner Support* latent variable, while “DDN_OH_A,” “N_SR_DES,” “P_OH_PB”, and “P_D_DES” were used as marker indicators for *Anxiety in Context of HCPs, Emotional Support from HCPs, Pain Behaviors in Context of Partner*, and *Distraction by Partner* latent variables, respectively. There were no double-loading indicators.

**Figure 1:**
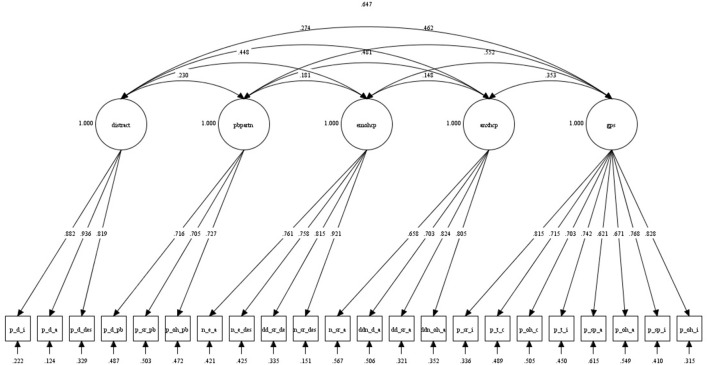
**Five-factor model derived from CFA in *N* = 120 individuals with musculoskeletal pain (Study 3)**. The figure shows completely standardized estimates which, if squared, correspond to percentage variance in observed measure accounted for by the latent factor. “gps” denotes *General Partner Support*, “anxhcp” *Anxiety in Context of HCPs*, “emohcp” *Emotional Support from HCPs*, “pbpartn” *Pain Behaviors in Context of Partner*, and “distract” denotes the *Distraction* by Partner factor. Indicator abbreviations are explained in Table 2.

Guidelines indicating acceptable model fit are presented in parentheses next to the results. For the 5-factor model, the chi-square test was significant, χ^2^_(199)_ = 308.42, *p* < 0.001, indicating that the null hypothesis that the model was a good fit should be rejected, and the TLI fell below the established cut-off, TLI = 0.92 (>0.95; Brown, 2006). However, other fit indices supported that model had an acceptable fit, RMSEA = 0.07 (<0.08; Browne and Cudeck, 1993), CFI = 0.93 (≥0.90; Browne and Cudeck, 1993), and SRMR = 0.07 (<0.08; Brown, 2006). Furthermore, all freely estimated unstandardized parameters were significant (*p*s < 0.001). For a summary of the model, see Figure 1. Indicators were moderately to strongly correlated with their latent factors (*R*^2^s = 0.39–0.88), demonstrating that the RASP items were reliable indicators of the five factors. By comparison, the more parsimonious 1-factor model did not fit the data at all: χ^2^_(212)_ = 926.33, *p* < 0.001; TLI = 0.48; RMSEA = 0.17; CFI = 0.52; SRMR = 0.18. Given the differences between the samples in Study 2 and the present study (different type of pain, different gender composition), the data from the CFA provides initial tentative support for the 5-factor model.

#### Construct validity

Cronbach's alphas were α = 0.938 for ECR-R anxiety and α = 0.943 for ECR-R avoidance, again demonstrating excellent reliabilities. As in Study 2, RASP scales were computed (see Table 4). Cronbach's alphas were α = 0.902 for *General Partner Support*, α = 0.884 for *Emotional Support from HCPs*, α = 0.832 for *Anxiety in Context of HCPs*, α = 0.753 for *Pain Behaviors in Context of Partner*, and α = 0.908 for *Distraction by Partner* scales. Partial correlations between RASP scales and attachment style dimensions were calculated (displayed in Table 5). Four participants did not complete the ECR-R, leaving *n* = 116 for this analysis. Mean ECR-R scores were *M* = 2.81 (*SD* = 1.21) for attachment anxiety (30–40th percentile; Fraley, R.C., personal communication, 2011) and *M* = 2.86 (*SD* = 1.26) for attachment avoidance (50–60th percentile). Therefore, this sample was more insecurely attached than the sample in Study 2. As in Study 2, *General Partner Support* and *Emotional Support from HCPs* scales were positively associated with attachment anxiety and negatively associated with attachment avoidance. However, in contrast to Study 2, no other correlations between RASP scales and attachment dimensions reached significance.

#### The relationship between sample characteristics and RASP scales

Results were very similar to those found in Study 2. Specifically, One-Way ANOVAs showed no differences between individuals with and without chronic pain [*F*_(1, 118)_ = 1.287, *p* = 0.259 for *General Partner Support*; *F*_(1, 118)_ = 0.619, *p* = 0.433 for *Emotional Support from HCPs*; *F*_(1, 118)_ = 1.113, *p* = 0.294 for *Anxiety in Context of HCPs*; *F*_(1, 118)_ = 1.479, *p* = 0.226 for *Pain Behaviors in Context of Partner*; *F*_(1, 118)_ = 3.328, *p* = 0.071 for *Distraction by Partner*], with and without depression [*F*_(1, 118)_ = 1.243, *p* = 0.267 for *General Partner Support*; *F*_(1, 118)_ = 0.05, *p* = 0.823 for *Emotional Support from HCPs*; *F*_(1, 118)_ = 2.370, *p* = 0.126 for *Anxiety in Context of HCPs*; *F*_(1, 118)_ = 2.062, *p* = 0.154 for *Pain Behaviors in Context of Partner*; *F*_(1, 118)_ = 0.239, *p* = 0.626 for *Distraction by Partner*], or between men and women [*F*_(1, 118)_ = 0.614, *p* = 0.435 for *General Partner Support*; *F*_(1, 118)_ = 0.032, *p* = 0.859 for *Emotional Support from HCPs*; *F*_(1, 118)_ = 0.482, *p* = 0.489 for *Anxiety in Context of HCPs*; *F*_(1, 118)_ = 1.103, *p* = 0.296 for *Pain Behaviors in Context of Partner*; *F*_(1, 118)_ = 1.633, *p* = 0.204 for *Distraction by Partner*] for any RASP scale scores.

Furthermore, participants again rated the intensity of their pain as moderate, and 69.2% of participants stated that their injury had occurred within the last year. Thus, the pain experience was both recent and meaningful. Importantly, 71.7% of participants had visited a HCP for their musculoskeletal pain, as opposed to only a third of participants in Study 2, which supported our choice of sample.

In the regression analyses (not carried out in Study 2 as scale scores there were derived from exploratory analyses), age emerged as the only significant predictor of *General Partner Support, Anxiety in Context of HCPs*, and *Distraction by Partner* scales, β = −0.337, *p* = 0.020; β = −0.411, *p* = 0.007; β = −0.387, *p* = 0.008, respectively. Thus, these scales became less endorsed with age (mirroring the results in Study 2), whereas the length of the relationship or relationship quality had no effect. For *Pain Behaviors in Context of Partner*, age was a significant predictor only at step 1, β = −0.260, *p* = 0.004, and none of the variables were significant predictors at step 2. Similarly, for *Emotional Support from HCPs*, age was a significant predictor at step 1, β = −0.284, *p* = 0.002, but at step 2 the only significant predictor was relationship happiness, β = −0.316, *p* = 0.020. The happier participants reported to be in their relationship, the less favorably they considered support from HCPs.

In sum, Study 3 provided initial confirmation of the 5-factor structure of the RASP in individuals with experiences of musculoskeletal pain. A fourth study was designed to provide further validation of the RASP and to broaden its applicability by examining the factor structure in a sample from the general population without a specific pain experience.

## Study 4: further validation of the RASP in a sample from the general population

### Aims

Study 4 aimed to (a) broaden the applicability of the RASP by recruiting individuals from the general population without a specific type of acute pain, (b) use confirmatory factor analysis (CFA) to replicate the 5-factor model fit in this sample, and (c) provide further validation of the RASP by demonstrating construct validity using three additional measures relating to coping and perceived social support.

### Measures

#### Responses and attitudes to support during pain questionnaire (RASP)

As in Study 3, the 22-item RASP was used.

#### Experiences in close relationships revised (ECR-R; Fraley et al., 2000)

See Study 2 for details. As participants in this study were recruited from Amazon Mechanical Turk, the ECR-R was included to assess the representativeness of the Amazon Mechanical Turk sample by comparing ECR-R scores and correlations with the RASP with those in Studies 2 and 3.

#### The pain catastrophizing scale (PCS; Sullivan et al., 1995)

The PCS is a 13-item measure of pain catastrophizing, assessing the tendency to exaggerate the threat value of anticipated or actual pain (e.g., “*It's terrible and I think it's never going to get any better*”), be pre-occupied with pain-related thoughts (e.g., “*I keep thinking about how much it hurts*”), and feel unable to cope with pain (e.g., “*There is nothing I can do to reduce the intensity of the pain*”). Items are rated on a scale ranging from 0 (*not at all*) to 4 (*all the time*), with higher scores denoting greater pain catastrophizing.

Pain catastrophizing has been both theoretically and empirically linked to the experience of pain within a social context. The communal coping model of pain catastrophizing (Sullivan et al., 2000) states that individuals who catastrophize about pain aim to solicit help and support from others to manage their pain. To this end, they communicate their need for assistance by engaging in displays of pain behaviors, such as wincing, moaning, or rubbing the painful area, and exhibiting general distress when in the presence of a potentially supportive person (Sullivan et al., 2001). Indeed, individuals with a higher tendency to catastrophize about pain engaged in more overt pain behaviors when another person was present than when they experienced pain alone (Sullivan et al., 2004). We thus expected PCS scores to be positively correlated with both the *Pain Behaviors in Context of Partner* and *Anxiety in Context of HCPs* RASP scales.

#### The multidimensional scale of perceived social support (MSPSS; Zimet et al., 1988)

The MSPSS is a 12-item measure of perceived social support from a significant other (4 items; e.g., “*There is a special person who is around when I am in need*”), family (4 items; e.g., “*My family really tries to help me*”), and friends (4 items; e.g., “*I have friends with whom I can share my joys and sorrows*”). Items are rated on a scale from 1 (*very strongly disagree*) to 7 (*very strongly agree*), with higher scores denoting greater perceived support. We hypothesized that MSPSS scores would be positively correlated with *General Partner Support, Distraction by Partner* and *Emotional Support from HCPs* scales, and that these associations would be strongest for the “significant other” subscale for the two partner-related RASP scales.

#### COPE inventory (Carver et al., 1989)

The COPE inventory comprises 11 facets of coping assessed by four-item subscales. For the purposes of this questionnaire validation, only the *Seeking social support for emotional reasons* scale was used. This scale captures the extent to which individuals seek emotional support from others (e.g., “*I try to get emotional support from friends or relatives*”), and was expected to be positively correlated with the *General Partner Support* and *Emotional Support from HCPs* RASP scales. Items are rated on a scale ranging from 0 (*I usually don't do this at all*) to 4 (*I usually do this a lot*); higher scores denote greater engagement in this coping strategy.

#### Items assessing the relationship with social support provider and demographic information

As in Studies 1–3, participants rated the quality of their relationship with their current partner (see Study 1 for details) and provided demographic information (see Study 2). This information was also used to assess the representativeness of the Amazon Mechanical Turk sample by examining participants' responses in this study in relation to Studies 1–3.

### Participants and procedure

Participants were recruited using the online crowdsourcing platform Amazon Mechanical Turk. Research has shown data from samples drawn from Amazon Mechanical Turk to be comparable to data collected via more traditional methods (e.g., Buhrmester et al., 2011; Mason and Suri, 2012; although see also Chandler et al., 2014, for potential problems with this recruitment method). To ensure fluency in English, participants were included if they lived in the United States of America. In addition, as in Studies 2 and 3, participants were required to be in a relationship to take part. The sample consisted of *N* = 180 individuals with a mean age of 33.75 years (*SD* = 10.13), of whom 51.7% were male and 48.3% were female (see Table 1 for comprehensive sample characteristics). Overall, the present sample was slightly older than the samples in Studies 1–3, which may explain why the average relationship length and chronic pain prevalence were slightly higher. Perceived empathy, closeness, and relationship happiness were very similar to Studies 1–3.

Participants completed the RASP, PCS, ECR-R, COPE, and MSPSS, always in this order, although the order of items within the questionnaires was randomized. As participants were not required to have experienced a certain type of pain to take part, they were asked to respond to the RASP in reference to acute back pain. We provided this instruction to ensure participants thought of the same type of pain. Back pain was chosen as this is a commonly experienced type of pain. Participants received $2.50 for their participation.

### Planned statistical analyses

The confirmatory factor analysis (CFA) strategy was identical to that employed in Study 3 with the exception that modification indices were requested and inspected to see whether allowing correlations amongst indicators loading onto the same factor would improve model fit. Construct validity was assessed by examining correlations between the RASP scales and the ECR-R (as in Studies 2 and 3) and three further measures, namely the PCS, MSPSS, and COPE.

### Results

#### Confirmatory factor analysis

No univariate or multivariate outliers were identified and thus all cases were included in the CFA. Five latent variables (*General Partner Support, Emotional Support from HCPs, Anxiety in Context of HCPs, Pain Behaviors in Context of Partner* and *Distraction by Partner*) were again specified to relate to the 22 RASP indicators. The same indicators as in Study 3 were used as marker indicators. Latent variables were allowed to be correlated and modification indices showed that model fit would be improved if items P_SP_A and P_SP_I (both on the *General Partner Support* scale) were allowed to correlate.

**Figure 2:**
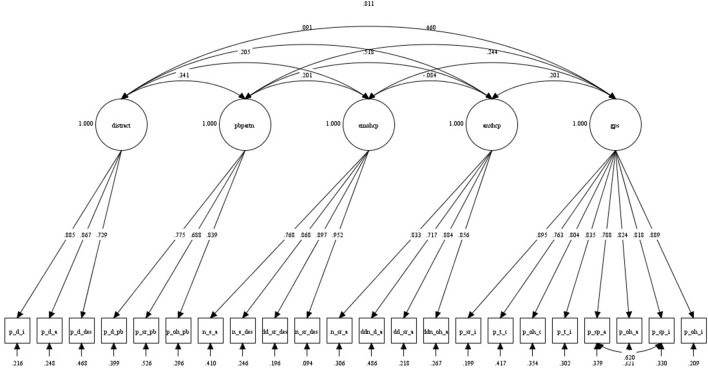
**Five-factor model derived from CFA in *N* = 180 individuals thinking of acute back pain (Study 4)**. The figure shows completely standardized estimates which, if squared, correspond to percentage variance in observed measure accounted for by the latent factor. “gps” denotes *General Partner Support*, “anxhcp” *Anxiety in Context of HCPs*, “emohcp” *Emotional Support from HCPs*, “pbpartn” *Pain Behaviors in Context of Partner*, and “distract” denotes the *Distraction* by Partner factor. Indicator abbreviations are explained in Table 2.

The guidelines indicating acceptable model fit are again presented in parentheses next to the results. As in Study 3, the chi-square test was significant, χ^2^_(198)_ = 390.55, *p* < 0.001 and the TLI fell below the established cut-off, TLI = 0.93 (>0.95; Brown, 2006). However, as in Study 3, the other fit indices supported that model had an acceptable fit, RMSEA = 0.07 (<0.08; Browne and Cudeck, 1993), CFI = 0.94 (≥0.90; Browne and Cudeck, 1993), and SRMR = 0.06 (<0.08; Brown, 2006). As in Study 3, all freely estimated unstandardized parameters were significant (*p*s < 0.001). For a summary of the model, see Figure 2. Compared to Study 3, indicators were more strongly correlated with their latent factors (*R*^2^s = 0.47–0.91), demonstrating again that the RASP items were reliable indicators of the five factors. As in Study 3, a more parsimonious 1-factor model did not fit the data at all: χ ^2^ (209) = 1716.95, *p* < 0.001; TLI = 0.47; RMSEA = 0.20; CFI = 0.52; SRMR = 0.17. Overall, the 5-factor model fit was very similar to the model fit in Study 3. This is encouraging given the differences between the samples. The present CFA thus provided further support for the 5-factor model.

#### Tests of construct validity

***RASP scales and adult attachment style***. Cronbach's alphas were α = 0.958 for ECR-R anxiety and α = 0.959 for ECR-R avoidance, again demonstrating excellent reliabilities. As in Studies 2 and 3, RASP scales were computed (see Table 4). RASP scale alphas were again good to excellent; α = 0.947 for *General Partner Support*, α = 0.925 for *Emotional Support from HCPs*, α = 0.893 for *Anxiety in Context of HCPs*, α = 0.805 for *Pain Behaviors in Context of Partner*, and α = 0.943 for *Distraction by Partner* scales. Partial correlations between RASP scales and attachment style dimensions were calculated (see Table 5). Three participants did not complete the ECR-R, leaving *n* = 177 for this analysis. Mean ECR-R scores were *M* = 2.53 (*SD* = 1.34) for attachment anxiety (20–30th percentile; Fraley, R.C., personal communication, 2011) and *M* = 2.57 (*SD* = 1.23) for attachment avoidance (40–50th percentile). Therefore, this sample was very similar in adult attachment style to the samples in Studies 2 and 3, supporting the representativeness of the Amazon Mechanical Turk sample.

As in Studies 2 and 3 and in line with our hypotheses, *General Partner Support* was positively associated with attachment anxiety and negatively associated with attachment avoidance (see Table 5). *Emotional Support from HCPs* was negatively associated with attachment avoidance. Further, as in Study 2 (but not Study 3), *Distraction by Partner* was positively associated with attachment anxiety and negatively associated with attachment avoidance, and *Anxiety in Context of HCPs* was positively associated with attachment anxiety. It is possible that results were more similar to Study 2 than 3 because ECR-R scores were also slightly more similar to those in Study 2. A surprising finding was the strong positive correlation between *Pain Behaviors in Context of Partner* and attachment anxiety, which was not found in Studies 2 and 3.

***Pain catastrophizing***. Cronbach's alpha for the PCS was α = 0.943 (mean score = 19.73, *SD* = 11.64). As predicted, significant positive correlations were found between pain catastrophizing and *Pain Behaviors in Context of Partner* (*r* = 0.239, *p* = 0.001) and *Anxiety in Context of HCPs* (*r* = 0.184, *p* = 0.013) scales, supporting the validity of the RASP. Although not explicitly hypothesized, pain catastrophizing was also significantly positively correlated with the *Emotional Support from HCPs* scale (*r* = 0.205, *p* = 0.006); the other correlations did not reach significance (*ps* > 0.05).

***Perceived social support***. Cronbach's alpha was α = 0.927 for the total MSPSS (mean score = 5.60, *SD* = 1.03), α = 0.958 for the significant other subscale (*M* = 6.08, *SD* = 1.14), α = 0.940 for the family subscale (*M* = 5.37, *SD* = 1.40), and α = 0.938 for the friends subscale (*M* = 5.34, *SD* = 1.29). As hypothesized, the total MSPSS score was significantly positively correlated with *General Partner Support* (*r* = 0.291, *p* < 0.001), *Emotional Support from HCPs* (*r* = 0.333, *p* < 0.001), and *Distraction by Partner* (*r* = 0.404, *p* < 0.001) RASP scales. Examining the subscales, these correlations were strongest for the significant other subscale, as anticipated (*r* = 0.349, *p* < 0.001, compared to *r* = 0.212 for family, and *r* = 0.160 for friends subscales for *General Partner Support; r* = 0.301, *p* < 0.001, compared to *r* = 0.260 for family, and *r* = 0.250 for friends subscales for *Emotional Support from HCPs*; *r* = 0.435, *p* < 0.001, compared to *r* = 0.309 for family, and *r* = 0.249 for friends subscales for *Distraction by Partner*). Correlations between MSPSS and *Anxiety in Context of HCPs* and *Pain Behaviors in Context of Partner* scales were not significant.

***Seeking emotional support***. Cronbach's alpha for the COPE *Seeking support for emotional reasons* scale was α = 0.882 (mean score = 10.96, *SD* = 3.22). As predicted, seeking emotional support was significantly positively correlated with *General Partner Support* (*r* = 0.464, *p* < 0.001) and *Emotional Support from HCPs* (*r* = 0.344, *p* < 0.001) scales. Further, seeking emotional support was also significantly positively correlated with *Distraction by Partner* (*r* = 0.447, *p* < 0.001) and *Pain Behaviors in Context of Partner* (*r* = 0.201, *p* = 0.007), while there was no relationship between this COPE scale and *Anxiety in Context of HCPs*.

In conclusion, Study 4 provided further validation of the RASP. The composition of the Amazon Mechanical Turk sample and the results were similar to Studies 2 and 3. The 5-factor model initially confirmed in Study 3 was replicated in this sample, and associations with measures assessing coping strategies and perceived social support further demonstrated the validity of the RASP in measuring attitudes and responses to social support in relation to pain.

## Discussion

We constructed and provided initial validation for a novel self-report questionnaire aimed to address the complex relationship between social support and acute pain in the general population. In four studies, we reported the development (Study 1) and psychometric evaluation (Studies 2–4) of the Responses and Attitudes to Support during Pain questionnaire (RASP). The final version of the RASP incorporates two frequent sources of social support, six facets of social support and five pain-related attitudes and responses. Specifically, it consists of 22 items representing five underlying dimensions: (1) *General Partner Support*, (2) *Emotional Support from HCPs*, (3) *Anxiety in Context of HCPs*, (4) *Pain Behaviors in Context of Partner*, and (5) *Distraction by Partner*. This 5-factor model was established in women with experience of menstrual pain who reported being in a relationship, and confirmation of this model was provided by individuals with recent experience of musculoskeletal pain (Study 3) and individuals referring to acute back pain (Study 4), who were also in a relationship. In all samples, the RASP scales showed good to excellent internal consistency. To our knowledge, the RASP is the only questionnaire to date dedicated to assessing attitudes and responses to social support in reference to acute bodily pain.

To avoid redundancy and ensure that the facets of social support were captured comprehensively in the items, there was a certain amount of terminological variability within and between the subscales. All items comprised three facets, namely source and type of support, and pain-related response or attitude, which were combined in different ways within each item. The separate items were phrased using semantically similar terms, e.g., “discomfort,” “unpleasant”, and “anxiety” were used to capture the general affective-motivational dimension of pain. The high internal consistency of the RASP subscales indicated that the items tapped into five distinguishable and coherent underlying components. In addition, these same components were found in two different samples (Studies 1 and 2; with the exception of the *Desirability of Partner Support* component) and confirmation of this component structure was provided in Studies 3 and 4.

To examine construct validity, the RASP was correlated with a measure of adult attachment style (Studies 2–4), and measures of pain catastrophizing, perceived social support, and coping by seeking emotional support (Study 4). We observed significant correlations between RASP scales and these measures in the expected directions, supporting the validity of the RASP. In addition, the validation constructs were differentially associated with the RASP scales (e.g., pain catastrophizing but not perceived social support was related to the *Pain Behaviors in Context of Partner* scale). Therefore, these results underline the need for a multifaceted measure specifically capturing individual differences in responding to support from others during pain, which we hope to have provided with the RASP.

Moreover, the psychometric evaluation of the RASP offered several insights into the social support and pain literature. First, a robust finding across studies (with the exception of the *Distraction* component in Study 1) was that partner and HCP items consistently loaded onto separate components. This underlines the need to distinguish between sources of social support (see also Christenfeld et al., 1997; Younger et al., 2010) over and above the type of support being assessed.

Second, while *General Partner Support* encompassed several types of social support, it is noteworthy that distraction by the partner comprised its own component, although it was correlated with *General Partner Support*. Distraction is not included in classical delineations of social support facets (Schaefer et al., 1981; Barrera, 1986) but has been recognized as an important feature of interpersonal interactions in pain, and shown to reduce pain in clinical (Manimala et al., 2000) and experimental studies (Jackson et al., 2005; Jackson, 2007). Thus, the RASP supports the importance of studying distraction by others as a separate social support facet.

Third, the RASP revealed interesting distinctions in attitudes and responses to social support from HCPs. At first sight, the two HCP scales might appear contradictory; one captured positive attitudes to empathy and reassurance from HCPs (doctors and nurses) and the other described anxiety when a range of support behaviors was provided by HCPs (predominantly doctors). Perhaps, the difference lies in the fact that the dominant feature of the *Emotional Support from HCPs* scale was the type of support, whereas the main characteristic of the *Anxiety in Context of HCPs* scale was the pain-related response. This suggests that the preferred type of support from HCPs and its actual effects on pain may dissociate. In addition, emotional support may be preferred from nurses, while doctors may be associated with more serious and more anxiety-provoking pain. Dakof and Taylor (1990) reported that 24% of cancer patients reported concern and affection from nurses as being helpful, followed by nurses being pleasant and kind (18%; Dakof and Taylor, 1990). For physicians, the expression of concern, empathy or affection was rated as helpful by only 11% of participants, and was mentioned in fourth place after informational support, competent medical care and optimism. A future questionnaire may need to assess these two sources of support separately (see also below).

Lastly, it is noticeable that empathy as a type of support featured chiefly in the HCP items. Indeed, only one partner empathy item was retained in the final measure, which loaded onto the *Pain Behaviors in Context of Partner* component. While the importance of reassurance and empathy from nurses is in line with previous research (Dakof and Taylor, 1990), it is thus possible that partner empathy is qualitatively different and linked more to solicitousness (Issner et al., 2012) and reinforcement of pain behaviors than to a construct of emotional support. Further, in Study 2 we observed that relationship happiness was negatively associated with the *Pain Behaviors in Context of Partner* scale. It is possible that participants with low relationship quality have to engage in more pain expression to secure adequate support, compatible with the communal coping model of pain catastrophizing which views pain behaviors as a means to solicit support (Sullivan et al., 2004). In this vein, we correlated the RASP with a measure of pain catastrophizing and found that pain catastrophizing was positively correlated with the *Pain Behaviors in Context of Partner* scale.

Several limitations of the current study warrant mention. Although we did not find any significant differences between individuals with or without chronic pain or depression in Studies 2 and 3, the proportion of individuals with chronic and depression was small. Therefore, conclusions regarding the applicability of the RASP in such populations vs. the general population may be premature, and future research including larger samples of individuals with chronic pain and depression is needed. In addition, although all participants in Studies 2–4 were in a relationship at the time of taking part, there were large variations in the length of the relationship. We therefore could not isolate the stage of the relationship (e.g., initial phase vs. established relationships), which may be important from an attachment theory perspective. Indeed, the RASP seems to apply best to individuals currently in a relationship. However, as 60% of women and 63% of men over the age of 16 were married or cohabiting in the UK in 2011 (Office for National Statistics, 2011), the RASP remains applicable to a large part of the population. Also, we observed that age was a significant predictor of *General Partner Support, Anxiety in Context of HCPs*, and *Distraction by Partner* scores. In future studies, it would be interesting to explore why increasing age seems to be linked to less strong attitudes to partner support and reduced anxiety in response to support from HCPs. Further, it would have been useful to include a larger test-retest sample. Lastly, in order to limit the number of items, several HCP items combined doctors, dentists and nurses, so that we were unable to assess whether one type of HCP was especially important in these items.

Our study also had a number of strengths. The gradual specification and development of the questionnaire, including asking participants about a specific type of pain in Studies 2 and 3 and including only participants in current relationships, as well as maximizing the exposure to HCPs (in Study 3), reduced variability in the data and ensured that participants could respond meaningfully to all items. Participants provided moderate pain intensity ratings in Studies 2 and 3, indicating that that they were referring to a relevant pain experience when completing the RASP. Moreover, although Studies 2 and 3 differed in terms of type of pain (menstrual pain vs. musculoskeletal pain; normal process vs. injury) and demographic factors (gender), the five-component model derived from Study 2 had an acceptable fit in Study 3, highlighting the utility of this measure in relatively different acute pain samples. Indeed, broadening the focus again to include participants not currently in pain (Study 4) and replicating the model fit in this sample indicates the applicability of the RASP in diverse populations, including individuals thinking about—but not currently experiencing—pain.

Furthermore, we combined theoretical approaches in guiding the RASP construction and thus hope the RASP will be applicable to a range of research questions and allow novel insights particularly into the social modulation of acute pain. Acute pain differs from chronic pain in its duration and thus arguably also in terms of patterns of social interactions which develop over time. Indeed, the measures relating to chronic pain mentioned in the introduction reflect theoretical perspectives such as operant conditioning (Fordyce, 1976), in which others' solicitous responses are seen to reinforce pain behaviors over time. This perspective may be less well suited to acute pain, and therefore a measure capturing facets related more directly to acute pain, such as the present RASP questionnaire, is needed. It would be an important future step to compare samples with acute and chronic pain and administer both the RASP and a measure suited to chronic pain to test that these measures indeed fit their respective populations best. Lastly, given the good to excellent internal consistency of the RASP scales, it would also be possible to administer only the partner or HCP scales depending on the sample and research question.

In conclusion, the RASP presents a multidimensional yet concise measure of attitudes and responses to social support in relation to acute pain, with good to excellent internal consistency, and good construct validity and test-retest reliability. Future studies could corroborate the psychometric properties of the questionnaire, particularly in relation to population characteristics such as gender and age. Further validation of the RASP in other acute pain samples, possibly immediately after a painful experience in the context of HCPs (such as wisdom tooth removal), would also be an important goal. The RASP is envisaged to be a useful methodological tool for assessing individual differences in diverse populations and ultimately for tailoring social support provision to the specific needs of individuals in pain.

## Author contributions

All authors contributed substantially to the manuscript and provided feedback on the draft paper. Charlotte Krahé carried out the research reported and wrote the paper; Yannis Paloyelis advised and assisted with statistical analyses for all studies; Chiara F. Sambo was involved in the conception of the research and the design and data collection for Study 1; and Aikaterini Fotopoulou was involved in the conception, design and interpretation of the results for all studies.

### Conflict of interest statement

The authors declare that the research was conducted in the absence of any commercial or financial relationships that could be construed as a potential conflict of interest.
